# Allogeneic stem cell transplantation may overcome the adverse impact of myelofibrosis on the prognosis of myelodysplastic syndrome

**DOI:** 10.1186/s40164-021-00238-x

**Published:** 2021-08-14

**Authors:** Xiangzong Zeng, Li Xuan, Zhiping Fan, Yu Zhang, Ke Zhao, Ya Zhou, Jun Xu, Qifa Liu, Min Dai

**Affiliations:** Department of Hematology, Nanfang Hospital, Southern Medical University, 1838 Guangzhou Blvd North, Guangzhou, China

**Keywords:** Myelodysplastic syndrome, Myelofibrosis, Prognosis, Hematopoietic cell transplantation

## Abstract

**Purpose:**

Myelofibrosis (MF) may serve as a poor prognostic factor in myelodysplastic syndromes (MDS). This study explored the impact of allogeneic hematopoietic stem cell transplantation (allo-HSCT) on the outcome of MDS patients with MF.

**Patients and Methods:**

Three hundred and sixteen MDS patients were enrolled in this retrospective study. Based on the degree of MF, we divided the patients into 2 groups: grade 0–1 (MF-0/1) and grade 2–3 (MF-2/3) groups. The clinical features, treatments, and prognosis in MDS patients with MF were analyzed.

**Results:**

Forty-three (13.6%) patients were diagnosed as MF-2/3. Complex karyotypes were more common in the MF-2/3 compared to MF-0/1 groups (*P* = 0.002). The overall response rate (ORR) of cytoreduction was 49.0%, along with 53.3% in the MF-0/1 and 16.7% in MF-2/3 groups (*P* = 0.017). In total, 141 patients underwent allo-HSCT, including 121 in the MF-0/1 and 20 in MF-2/3 groups. The median time to neutrophil reconstruction was 12 (range: 7–34) and 14 (range: 10–45) days (*P* = 0.005), and platelet reconstruction was 14 (range: 8–68) and 18 (range: 8–65) days (*P* = 0.045) in the MF-0/1 and MF-2/3 groups, respectively. However, the cumulative incidence of neutrophil and platelet engraftment achieved at day + 30 was not different between the two groups (*P* = 0.107, *P* = 0.303, respectively). Non-relapse mortality, relapse, and acute and chronic graft-versus-host disease were similar between the two groups (all *P* > 0.05). Among patients with allo-HSCT, the 2-year overall survival (OS) was 68.5% (95% CI: 60.1–76.9%) and 68.4% (95% CI: 47.4–89.4%) in the MF-0/1 and MF-2/3 groups, respectively, (*P* = 0.636). Among patients without allo-HSCT, the 2-year OS was 49.9% (95% CI: 40.7–59.1%) and 19.2% (95% CI: 0–39.6%) in the MF-0/1 and MF-2/3 groups, respectively, (*P* = 0.002). In multivariate cox analysis, complex karyotype was an unfavorable factor for relapse (HR, 4.16; *P* = 0.006), disease-free survival (DFS) (HR, 2.16; *P* = 0.020), and OS (HR, 2.47; *P* = 0.009) post-transplantation.

**Conclusion:**

Patients with MF-2/3 have more complex karyotypes and lower ORR of cytoreduction in MDS. Among patients without allo-HSCT, patients with MF-2/3 have a worse prognosis than those with MF-0/1. However, the adverse impact of MF on prognosis may be overcome by allo-HSCT.

## Introduction

Myelodysplastic syndromes (MDSs) are a group of myeloid clonal hemopathies with a relatively heterogeneous spectrum of presentation, characterized by varying degrees of cytopenias and a predisposition to acute myeloid leukemia (AML) [1]. Several scoring systems, such as International Prognostic Scoring System (IPSS) and its revised version (IPSS-R), have been developed to stratify MDS. However, these scoring systems depend mainly on the percentage of the myeloblasts, karyotypic abnormalities, and the number of peripheral blood cytopenias [2]. Myelofibrosis (MF) is observed in approximately 13–35% of MDS patients [3–5]. Increasing evidence suggests that MF is an adverse factor in MDS, which is associated with early bone marrow failure and shorter survival [3, 6, 7], although this risk feature is not captured by IPSS-R.

MF is characterized by variable degrees of cytopenias, a leukoerythroblastic blood picture, and extramedullary hematopoiesis resulting in progressive splenomegaly and debilitating disease-related constitutional symptoms, compromising quality of life [8–10]. MF can present as primary myelofibrosis (PMF), or accompanied by / arise from a pre-existing diagnosis of MDS, polycythemia vera or essential thrombocythemia and so on [11]. The most important difference between PMF and MDS with MF is the apparent multilineage lineage dysplasia observed in the latter [12]. What’s more, there are less heteromorphic and broken erythrocytes and there is no obvious hepatosplenomegaly in MDS with MF [13]. There are a group of diseases shares both dysplastic and proliferative features at the time of initial presentation which has been recognized as Myelodysplastic/Myeloproliferative Neoplasms (MDS/MPN), and includes chronic myelomonocyic leukaemia (CMML), atypical chronic myeloid leukaemia (aCML), juvenile myelomonocytic leukaemia (JMML) and MDS/MPN unclassified (MDS/MPN-U) [12]. These diseases can also present with cytopenias, MDS-like blood cell dysplasia and MF, but they are often accompanied by prominent myeloproliferative features, such as persistent peripheral blood monocytosis > 1 × 10^9^/L, increased proliferation of neutrophils or platelets and splenomegaly and so on [14]. At the molecular level, MDS/MPN disorders are more likely to carry gene mutations associated with the activation of growth factor signaling pathways in conjunction with mutations in epigenetic regulators or splicing factors associated with morphologic dysplasia [15–18]. In this study, we only enrolled patients with MDS with MF for analysis.

Allogeneic hematopoietic stem cell transplantation (allo-HSCT) is a unique curative option for MDS. Whether MF affects the outcomes post-transplantation remains controversial. Some studies suggested that MF was not an adverse factor on survival following allo-HSCT [19]. Still, other studies were suggesting MF might affect survival, especially those with moderate-to-severe MF [3, 5, 7, 20]. In this study, we aimed to explore the impact of MF on MDS outcomes. We retrospectively analyzed the clinical features, treatments, and prognosis in MDS patients with MF, as well as the main events post-transplantation, such as engraftment, graft-versus-host disease (GVHD), and survival.

## Patients and methods

### Study design and patients

This retrospective study cohort collected all primary MDS patients with known bone marrow histology at diagnosis from October 2012 to June 2019 at our institution. Patients with PMF or overlap syndromes, such as MDS/MPN, were excluded from the study. Details of follow-up data came from medical records and telephone follow-up. Diagnostic criteria were based on the World Health Organization (WHO) [12], while risk stratification was categorized according to the IPSS-R [2], and response evaluation was performed according to the International Working Group (IWG) [21].

### Cytogenetics and myelofibrosis assessment

Bone marrow (BM) specimens were used for cytogenetic analysis by conventional chromosome banding techniques and/or fluorescence in situ hybridization. Twenty metaphases were analyzed, and the results were reported using the current International System for Human Cytogenetic Nomenclature [22].

Sections of formalin-fixed and paraffin-embedded BM biopsies were stained with hematoxylin and eosin for morphology studies. The sections were also stained with Gomori silver-impregnation method to assess the reticulin fibers, amount of collagen deposition, and degree of osteosclerosis. According to the European MF network criteria [23], MF-0 is defined as “scattered linear reticulin with no intersections (crossovers) corresponding to normal BM”. MF-1 is defined as a “loose network of reticulin with many intersections, especially in perivascular areas”. MF-2 is defined as “diffuse and dense increase in reticulin with extensive intersections, occasionally with focal bundles of thick fibers mostly consistent with collagen, and/or focal osteosclerosis”. MF-3 is defined as “diffuse and dense increase in reticulin with extensive intersections and coarse bundles of thick fibers consistent with collagen, usually associated with osteosclerosis”. Cases with MF-2/3 were considered to be moderate-to-severe MF. Patients were diagnosed as “MDS with MF” when they meeted the diagnostic criteria of MDS and BM biopsy indicated MF > 0, in the absence of other prominent myeloproliferative features.

### Treatment algorithm

Based on the clinical practice guidelines [1, 24–26] for MDS, patients with lower-risk MDS were treated with either immunoregulatory therapy (including lenalidomide and cyclosporine) or supportive care alone (including transfusion, erythropoiesis-stimulating agents and iron chelation). If patients had transfusion dependence, they would be given hypomethylating agents (HMA) or allo-HSCT when appropriate donors existed. The patients with higher-risk MDS were treated with allo-HSCT if they were candidates for the treatment and had the appropriate donors, with or without pre-transplantation cytoreductive treatments (including HMA, chemotherapy combined with HMA, and traditional AML-like chemotherapy). If they were not suitable for HSCT, they would be given cytoreductive treatments or only supportive care according to their physical condition [27–29].

### Definitions and statistical analysis

Overall survival (OS) was calculated from the date of diagnosis to the date of death. By contrast, OS post-transplantation was calculated from the date of transplantation to the date of death. Surviving patients were censored at the date on which they were last known to be alive. Disease-free survival (DFS) was calculated from the time of transplantation to the date of relapse or death and was censored at the last date known to be alive and disease-free. Non-relapse mortality (NRM) was defined as death due to any cause without relapse. The overall response rate (ORR) was expressed as the percentage of patients achieving complete remission (CR) (including marrow CR), partial remission, or hematology improvement.

Comparison of numerical variables between groups was carried out using a Student’s t-test or a nonparametric approach (Mann–Whitney test). Comparing the distribution of categorical variables in different groups was performed with either Fisher’s exact test (2 × 2 tables) or the *χ*^2^ test (larger tables). Curves were constructed for OS using the Kaplan–Meier method and compared using a log-rank test (if no competing risk were involved) or computed in a competing risk framework by the method of Fine and Gray. Univariable and multivariable analyses were performed using a Cox proportional hazards regression model. *P* values were two-sided and considered significant if less than 0.05. SPSS 21.0 (SPSS, Inc, Chicago, Illinois) and R version 4.0.3 (R Development Core Team, Vienna, Austria) were used for data analysis.

## Results

### Patients’ baseline and treatment characteristics

A total of 316 hospitalized patients with newly diagnosed MDS were enrolled in this study. There were 108 females and 208 males, with a median age of 52 (range: 14–83) years. Finally, 43 (13.6%) patients were diagnosed as MF-2/3, including 28 MF-2 and 15 MF-3. Based on the degree of MF, we divided the patients into 2 groups: MF-0/1 and MF-2/3 groups. The patients’ baseline characteristics at diagnosis and treatments are detailed in Table 1, which show no differences except for the cytogenetics between the two groups (all *P* > 0.05). Aberrant karyotypes were more common in the MF-2/3 than the MF-0/1 group (*P* = 0.001). Further analysis showed complex karyotype was different between the two groups (*P* = 0.002). At the same time, no significant statistical differences were observed in + 8, -7, -Y, del(20q), del(7q), del(5q), double including del(5q-), double including -7/del(7q), and i(17q) between the two groups (all *P* > 0.05). In the MF-2/3 group, the baseline characteristics at diagnosis were compared between patients treated with allo-HSCT and those without allo-HSCT. The results showed patients treated with allo-HSCT were yonger than those without allo-HSCT (*P* = 0.003) (Table 2).

**Table 1:** The patients’ characteristics at diagnosis according to myelofibrosis (n = 316)

	MF = 0/1 (n = 273)	MF = 2/3 (n = 43)	*P*
Age (years), median, range	52 (14–83)	52 (21–79)	0.705
Gender, no. (%)			
Male	181 (66.3%)	27 (62.8%)	0.652
Female	92 (33.7%)	16 (37.2%)	
Median WBC (range), × 10^9^/L	2.66 (0.27–13.47)	2.73 (1.10–12.00)	0.609
Median NE (range), × 10^9^/L	0.99 (0.02–11.34)	0.85 (0.04–4.92)	0.794
Median HGB (range), g/L	69 (26–150)	67 (30–138)	0.291
Median PLT (range), × 10^9^/L	53 (1–408)	47 (3–282)	0.911
MDS subtypes (WHO, 2016), no. (%)			0.243
MDS-SLD	11 (4.0%)	2 (4.7%)	
MDS-MLD	52 (19.1%)	4 (9.3%)	
MDS-RS-SLD	2 (0.7%)	2 (4.7%)	
MDS-RS-MLD	8 (2.9%)	3 (7.0%)	
MDS with isolated 5q-deletion	2 (0.7%)	0 (0.0%)	
MDS-EB-1	70 (25.7%)	10 (23.2%)	
MDS-EB-2	118 (43.2%)	21 (48.8%)	
MDS-U	10 (3.7%)	1 (2.3%)	
Cytogenetic			0.001
Normal karyotype	148 (54.2%)	12 (27.9%)	
Aberrant karyotype	125 (45.8%)	31 (72.1%)	
IPSS-R risk group, no. (%)			0.859
Very low	4 (1.5%)	0 (0.0%)	
Low	29 (10.6%)	5 (11.6%)	
Intermediate	65 (23.8%)	9 (20.9%)	
High	84 (30.8%)	12 (27.9%)	
Very high	91 (33.3%)	17 (39.6%)	
Treatment, no. (%)			0.170
Allogeneic HSCT	121 (44.3%)	20 (46.5%)	
Cytoreductive treatments without HSCT	90 (33.0%)	12 (27.9%)	
HMA	68 (25.0%)	10 (23.2%)	
Chemotherapy combined with HMA	20 (7.3%)	2 (4.7%)	
AML-like chemotherapy	2 (0.7%)	0 (0.0%)	
Immunoregulatory	11 (4.0%)	5 (11.6%)	
Supportive care	51 (18.7%)	6 (14.0%)	
Leukemia transformation, no. (%)	50 (18.3%)	8 (18.6%)	0.964
Median time from diagnosis to leukemia transformation, months (range)	12 (2–31)	7 (1–20)	0.043

*MF* myelofibrosis*, WBC* white blood cell count, *NE* neutrophils, *HGB* hemoglobin, *PLT* platelet, *MDS-SLD* MDS with single-lineage dysplasia, *MDS-MLD* MDS with ring sideroblasts with multilineage dysplasia, *MDS-RS-SLD* MDS with ring sideroblasts with single-lineage dysplasia, *MDS-RS-MLD* MDS with ring sideroblasts with multilineage dysplasia, *MDS-EB* MDS with excess of blasts, *MDS-U* MDS unclassifiable, *HSCT* hematopoietic stem cell transplantation, *HMA* hypomethylating agents, *AML* acute myeloid leukemia

**Table 2:** The baseline characteristics at diagnosis of MDS patients with MF-2/3 (n = 43)

	Transplant (n = 20)	Non-transplant (n = 23)	P
Age (years), median, range	42 (27–60)	58 (21–79)	0.003
Gender, no. (%)			0.106
Male	10 (50.0%)	17 (73.9%)	
Female	10 (50.0%)	6 (26.1%)	
Median WBC (range), × 10^9^/L	2.73 (1.10–12.00)	2.91 (1.34–7.36)	0.759
Median NE (range), × 10^9^/L	0.85 (0.04–2.27)	0.92 (0.16–4.92)	0.439
Median HGB (range), g/L	65 (30–138)	69 (36–84)	0.706
Median PLT (range), × 10^9^/L	41.5 (3–234)	82 (8–282)	0.179
MDS subtypes (WHO, 2016), no. (%)			0.290
MDS-SLD	2 (10.0%)	0 (0.0%)	
MDS-MLD	3 (15.0%)	1 (4.3%)	
MDS-RS-SLD	0 (0.0%)	2 (8.7%)	
MDS-RS-MLD	1 (5.0%)	2 (8.7%)	
MDS with isolated 5q-deletion	0 (0.0%)	0 (0.0%)	
MDS-EB-1	5 (25.0%)	5 (21.8%)	
MDS-EB-2	8 (40.0%)	13 (56.5%)	
MDS-U	1 (5.0%)	0 (0.0%)	
Cytogenetic			0.692
Normal karyotype	5 (25.0%)	7 (30.4%)	
Aberrant karyotype	15 (75.0%)	16 (69.6%)	
IPSS-R risk group, no. (%)			0.128
Very low	0 (0.0%)	0 (0.0%)	
Low	2 (10.0%)	3 (13.0%)	
Intermediate	7 (35.0%)	2 (8.7%)	
High	6 (30.0%)	6 (26.1%)	
Very high	5 (25.0%)	12 (52.2%)	
Treatment, no. (%)			
Cytoreductive treatments pre-transplantation	11 (55.0%)	0 (0.0%)	
Cytoreductive treatments without HSCT	0 (0.0%)	12 (52.2%)	
Immunoregulatory	0 (0.0%)	5 (21.7%)	
Supportive care	0 (0.0%)	6 (26.1%)	
Leukemia transformation, no. (%)	6 (30.0%)	2 (8.3%)	0.115
Median time from diagnosis to leukemia transformation, months (range)	7 (1–20)	12 (9–15)	0.249

In total, 102 patients received cytoreductive treatments without allo-HSCT, which included 78 patients receiving HMA, 22 patients receiving chemotherapy combined with HMA, and 2 patients receivving traditional AML-like chemotherapy (Table 1). The other 141 patients received allo-HSCT (including 66 without cytoreduction and 75 with cytoreduction pre-transplantation), 16 patients received immunoregulatory, and 57 patients received only supportive care. There was no significant difference in the treatments between the two groups (*P* = 0.170) (Table 3).

**Table 3:** The patients’ characteristics at transplantation according to myelofibrosis (n = 141)

	MF = 0/1 (n = 121)	MF = 2/3 (n = 20)	*P*
Median age of recipient at HSCT (range)	44 (18–66)	42 (27–60)	0.747
Recipient sex			0.511
Male	70 (57.9%)	10 (50.0%)	
Female	51 (42.1%)	10 (50.0%)	
Recipient cytogenetic			0.743
Non-complex karyotypes	102 (84.3%)	16 (80.0%)	
Complex karyotypes	19 (15.7%)	4 (20.0%)	
BM blasts at HSCT			0.016
≤ 5%	29 (24.0%)	10 (50.0%)	
> 5%	92 (76.0%)	10 (50.0%)	
Time to HSCT from diagnosis			0.529
≤ 6 months	87 (71.9%)	13 (65.0%)	
> 6 months	34 (28.1%)	7 (35.0%)	
Previous therapy for MDS			0.861
No cytoreductive treatments	57 (47.1%)	9 (45.0%)	
Cytoreductive treatments	64 (52.9%)	11 (55.0%)	
HMA	27 (22.3%)	4 (20.0%)	
Chemotherapy combined with HMA	34 (28.1%)	7 (35%)	
AML-like chemotherapy	3 (2.5%)	0 (0.0%)	
Median age of donor (range)	37 (10–63)	40 (26–60)	0.181
Donor sex			0.323
Male	77 (63.6%)	15 (75.0%)	
Female	44 (34.2%)	5 (25.0%)	
Donor source			0.838
HID	51 (42.1%)	8 (40%)	
MUD	10 (8.3%)	1 (5%)	
MSD	60 (49.6%)	11 (55%)	
Stem cell source			0.605
PB	62 (51.2%)	9 (45.0%)	
PB + BM	59 (48.8%)	11 (55.0%)	
Median mononuclear cells per graft × 10^8^/kg, (range)	10.2 (7.21–13.9)	10.4 (8.3–14.0)	0.479
Median CD34 + cells per graft × 10^6^/kg, (range)	9.5 (6.8–13.1)	9.8 (8.1–13.7)	0.246
Conditioning regimens			0.051
BUCY	56 (46.3%)	3 (15.0%)	
DAC + BUCY	48 (39.7%)	13 (65.0%)	
BUCY + others	9 (7.4%)	3 (15.0%)	
BF	8 (6.6%)	1 (5.0%)	
GVHD prophylaxis			0.870
CsA + MTX + MMF	60 (49.6%)	11 (55.0%)	
CsA + MTX + ATG	5 (4.1%)	1 (5.0%)	
CsA + MTX + ATG + MMF	56 (46.3%)	8 (40.0%)	

*BM* bone marrow, *HID* haploidentical donor, *MUD* matched unrelated donor, *MSD* HLA-matched sibling donor, *PB* peripheral blood, *BUCY* busulfan + cyclophosphamide, *DAC* decitabine, *BF* busulfan + fludarabine; *GVHD* graft-versus-host disease, *CsA* cyclosporin A, *MTX* methotrexate, *MMF* mycophenolate, *ATG* antithymocyte globulin

### Leukemia transformation

With a median follow-up of 30 months (range: 1–142) post-diagnosis, 58 (18.4%) patients developed leukemia, including 50 (18.3%) cases in the MF-0/1 and 8 (18.6%) in the MF-2/3 group (*P* = 0.964) (Table1). The median transformation time was 11 months (range: 1–31), along with 12 months (range: 2–31) in the MF-0/1 and 7 months (range: 1–20) in the MF-2/3 group (*P* = 0.043).

### Cytoreductive treatments and outcomes

Of the 102 patients who received cytoreductive treatments without allo-HSCT, there were 90 cases in the MF-0/1, including 68 HMA and 20 chemotherapy combined with HMA and 2 traditional AML-like chemotherapy, and 12 in the MF-2/3 group, including 10 HMA and 2 chemotherapy combined with HMA. The ORR of cytoreduction was 49.0%, along with 53.3% in the MF-0/1 and 16.7% in the MF-2/3 group (*P* = 0.017) (Table 4), with a median cycle of 5 (range: 1–10) and 3 (range: 1–7), respectively (*P* = 0.111).

**Table 4:** The evaluation of the response to cytoreductive trearments in MDS patients (n = 102)

Response	MF-0/1 (n = 90)	MF-2/3 (n = 12)	P
ORR	48 (53.3%)	2 (16.7%)	0.017
CR	30 (33.3%)	1 (8.3%)	–
PR	11 (12.2%)	1 (8.3%)	–
HI	7 (7.8%)	0 (0.0%)	–
NR	42 (46.7%)	10 (83.3%)	–
SD	6 (6.7%)	2 (16.6%)	–
PD	36 (40.0%)	8 (66.7%)	–

*ORR* overall response rates*, CR* complete remission (including marrow CR), *PR* partial remission, *HI* hematological Improvement, *NR* no response, *SD* stable disease, *PD* progressive disease

Twenty-seven patients experienced relapse at a median time of 9 months (range: 1–35) after CR, including 26/30 cases in the MF-0/1 and 1/1 in the MF-2/3 group. The cumulative incidence of relapse at 2 years after CR was 88.3% (95% CI: 65.9–96.4%).

### Transplantation and outcomes

Of the 141 patients who received allo-HSCT, including 121 in the MF-0/1 and 20 in the MF-2/3 group, 71 patients received HLA-matched sibling donors (MSD) (MF-0/1, n = 60; MF-2/3, n = 11), 11 matched unrelated donors (MUD) (MF-0/1, n = 10; MF-2/3, n = 1), and 59 haploidentical donors (HID) (MF-0/1, n = 51; MF-2/3, n = 8) (*P* = 0.838). All patients received busulfan (BU)-based myeloablative conditioning (MAC) regimens as described previously [27]. Cyclosporin A (CsA) + methotrexate (MTX) + mycophenolate (MMF) were administered to patients undergoing MSD transplantation, and CsA + MTX + antithymocyte globulin (ATG) and/or MMF were administered to patients undergoing MUD or HID transplantation for GVHD prophylaxis [30]. The patients’ characteristics at transplantation are detailed in Table 3.

The cumulative incidence of neutrophil reconstruction (absolute neutrophil count > 0.5 × 10^9^/L) at day + 30 was 97.8% (95% confidence interval (CI): 92.8–99.4%), along with 98.3% (95% CI: 92.3–99.7%) in the MF-0/1 and 95.0% (95% CI: 53.0–99.6%) in the MF-2/3 group (*P* = 0.107). The median time to neutrophil reconstruction was 12 (range: 7–34) and 14 (range: 10–45) days in the MF-0/1 and MF-2/3 groups, respectively (*P* = 0.005) (Table 5). The cumulative incidence of platelet reconstruction (platelet > 20 × 10^9^/L) at day + 30 was 86.5% (95% CI: 79.6–91.2%), along with 87.6% (95% CI: 80.1–92.4%) in the MF-0/1 and 80.0% (95% CI: 52.8–92.5%) in the MF-2/3 group (*P* = 0.303). The median time to platelet reconstruction was 14 (range: 8–68) and 18 (range: 8–65) days in the MF-0/1 and MF-2/3 groups, respectively (*P* = 0.045) (Table 5).

**Table 5:** Results of stem cell transplantation in MDS patients with MF = 0/1 or MF = 2/3 (n = 141)

	MF = 0/1) (n = 121)	MF = 2/3 (n = 20)	*P*
Cumulative incidence of neutrophil reconstruction at day + 30	98.3%	95.0%	0.107
Cumulative incidence of platelet reconstruction at day + 30	87.6%	80.0%	0.303
Median days to neutrophil reconstruction(> 0.5 × 10^9^/L) (range)	12(7–34)	14(10–45)	0.005
Median days to platelet reconstruction(> 20X10^9^/L) (range)	14(8–68)	18(8–65)	0.045
Acute GVHD grade II–IV	45(37.2%)	6(30%)	0.535
Chronic GVHD	67(55.4%)	10(50%)	0.655
2 year cumulative incidence of relapse	12.0%	16.8%	0.910
2 year non-replase mortality	26.0%	21.7%	0.441

Fifty-one (36.2%) patients were diagnosed with acute GVHD (grades II–IV), along with 45 (37.2%) in the MF-0/1 and 6 (30.0%) in the MF-2/3 group (*P* = 0.535). Seventy-seven (54.6%) patients were diagnosed with chronic GVHD, along with 67 (55.4%) in the MF-0/1 and 10 (50%) in the MF-2/3 group (*P* = 0.655) (Table 5).

**Fig. 1:**
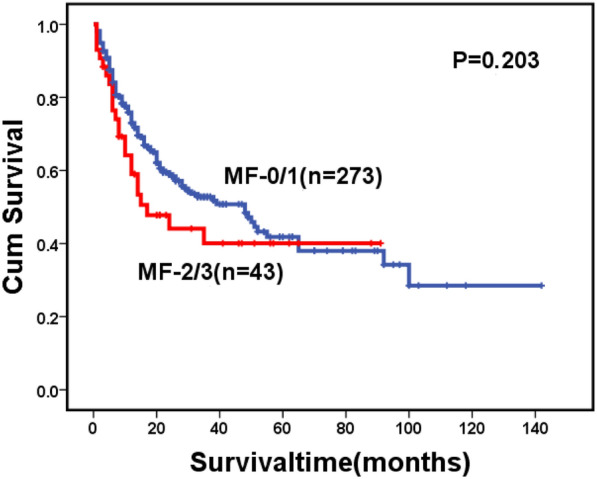
Overal survival of MDS patients classified by MF (MF-0/1 VS. MF-2/3)

**Fig. 2:**
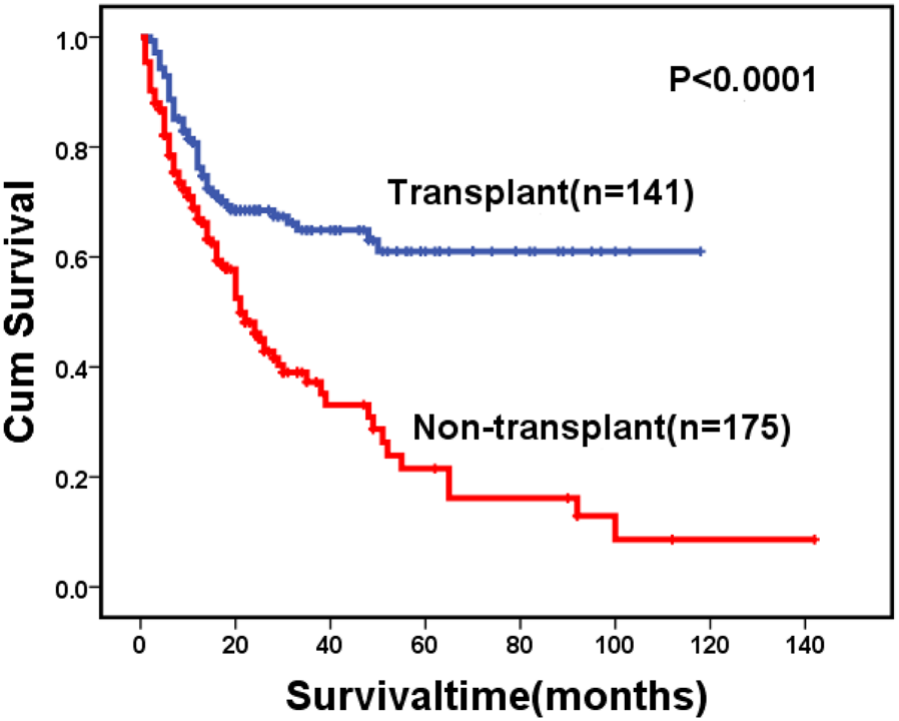
Overal survival of MDS patients classified by treatment (transplant VS. non-transplant)

**Fig. 3:**
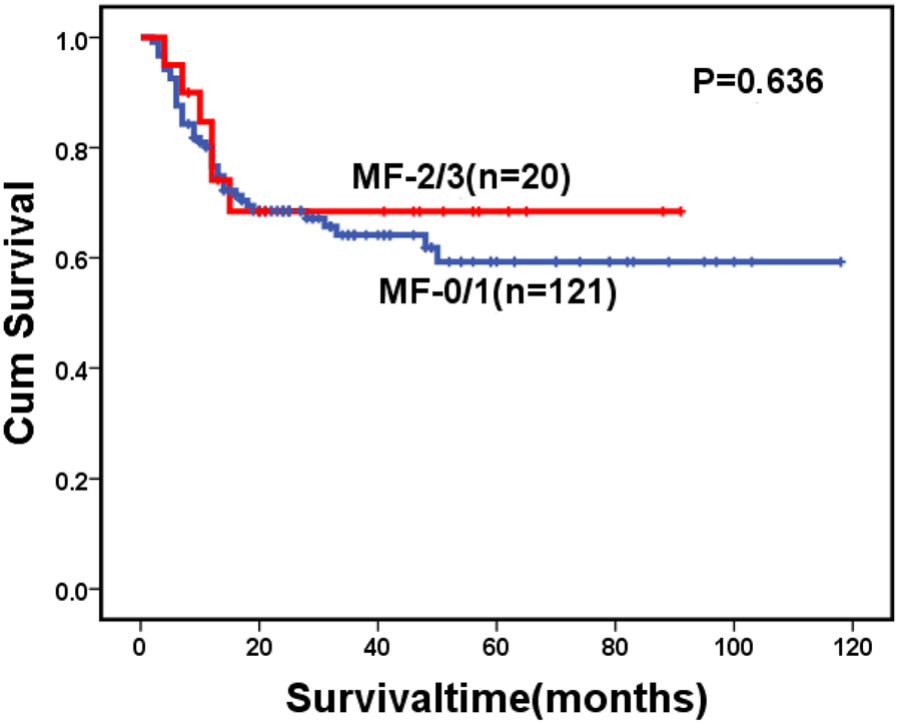
Overal survival of MDS patients with allo-HSCT, classified by MF (MF-0/1 VS. MF-2/3)

**Fig. 4:**
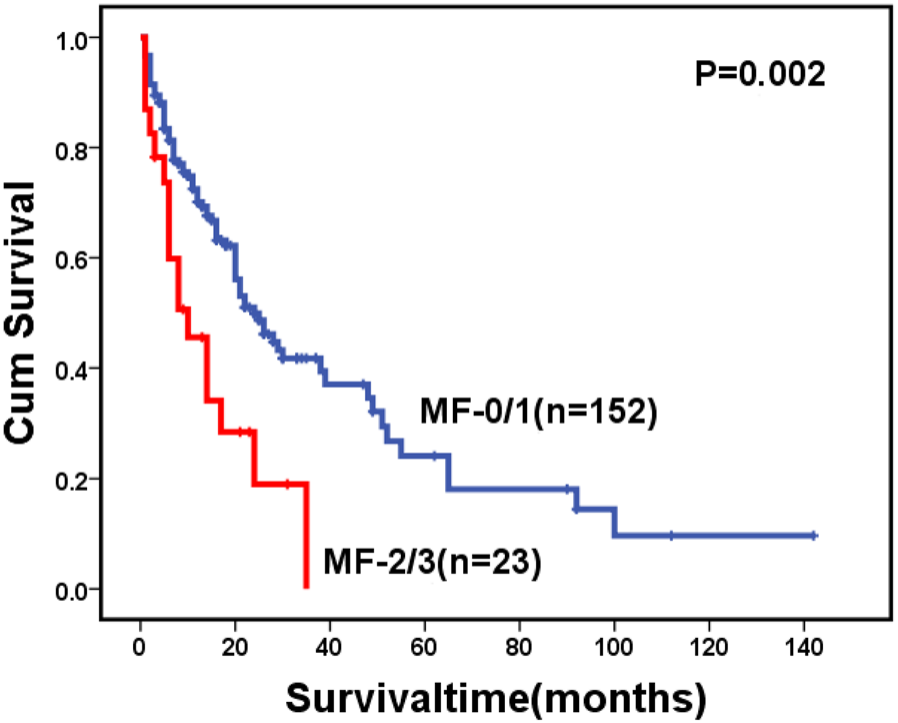
Overal survival of MDS patients without allo-HSCT, classified by MF (MF-0/1 VS. MF-2/3)

**Fig. 5:**
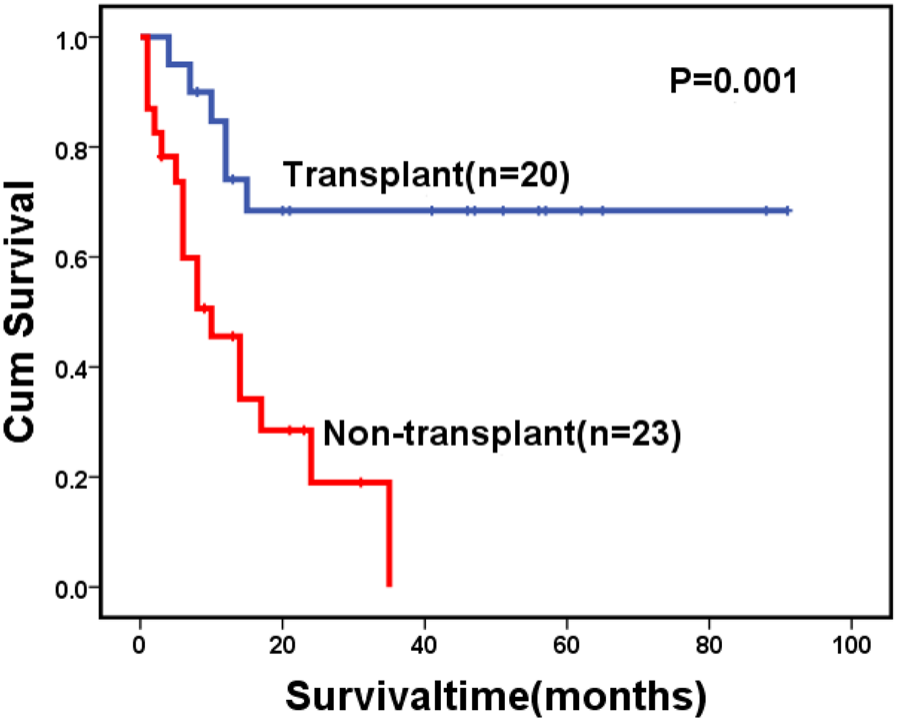
Overal survival of MDS patients with MF-2/3 classified by treatment (transplant VS. non-transplant)

For the whole cohort, 146 patients died during follow-up, including 123 cases in the MF-0/1 and 23 in the MF-2/3 group. The causes of death are shown in Table 6. The 2-year OS for the whole cohort was 57.1% (95% CI: 51.2–63.0%), along with 59.0% (95% CI: 52.7–65.3%) in the MF-0/1 and 53.6% (95% CI: 38.1–69.1%) in the MF-2/3 group (*P* = 0.211) (Fig. 1). The 2-year OS was 68.5% (95% CI: 60.7–76.3%) and 45.2% (95% CI: 36.8–53.6%) in the patients with and without allo-HSCT, respectively (*P* < 0.0001) (Fig. 2). Among patients with allo-HSCT, the 2-year OS was 68.5% (95% CI: 60.1–76.9%) and 68.4% (95% CI: 47.4–89.4%) in the MF-0/1 and MF-2/3 groups, respectively (*P* = 0.636) (Fig. 3). Among patients without allo-HSCT, the 2-year OS was 49.9% (95% CI: 40.7–59.1%) and 19.2% (95% CI: 0–39.6%) in the MF-0/1 and MF-2/3 groups, respectively (*P* = 0.002) (Fig. 4). The OS of patients with allo-HSCT was longer than those without allo-HSCT in the MF-2/3 group (*P* = 0.001) (Fig. 5).

**Table 6:** The causes of death(n = 146)

	MF = 0/1) (n = 123)	MF = 2/3 (n = 23)	*P*
Disease progression	52 (42.3%)	10 (43.5%)	0.324
Infections	24 (19.5%)	5 (21.7%)	
Bleeding	7 (5.7%)	4 (17.4%)	
Multiple organ failure	11 (8.9%)	1 (4.3%)	
Transplantation-related complications	10 (8.1%)	2 (8.7%)	
Others	19(15.4%)	1(4.3%)

**Fig. 6:**
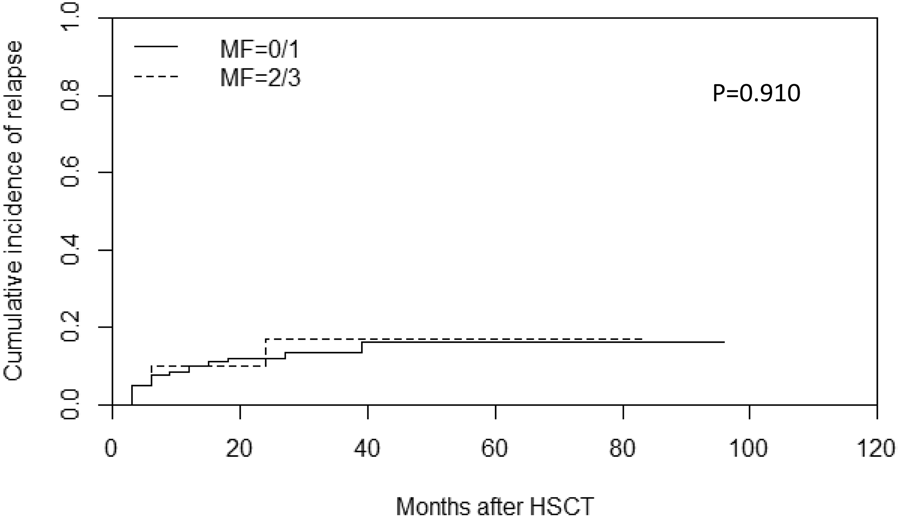
Cumulative incidence of relapse classified by MF (MF-0/1 VS. MF-2/3)

**Fig. 7:**
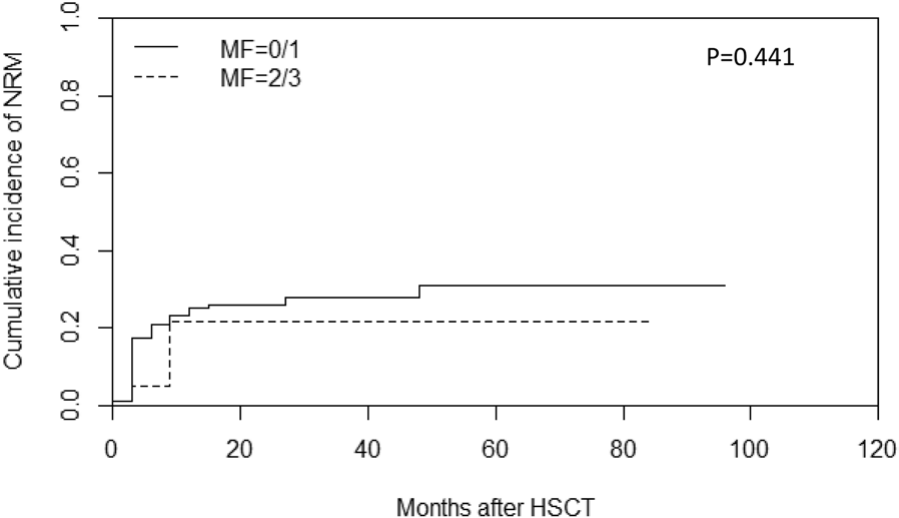
Cumulative incidence of non-replase mortality (NRM) classified by MF (MF-0/1 VS. MF-2/3)

**Fig. 8:**
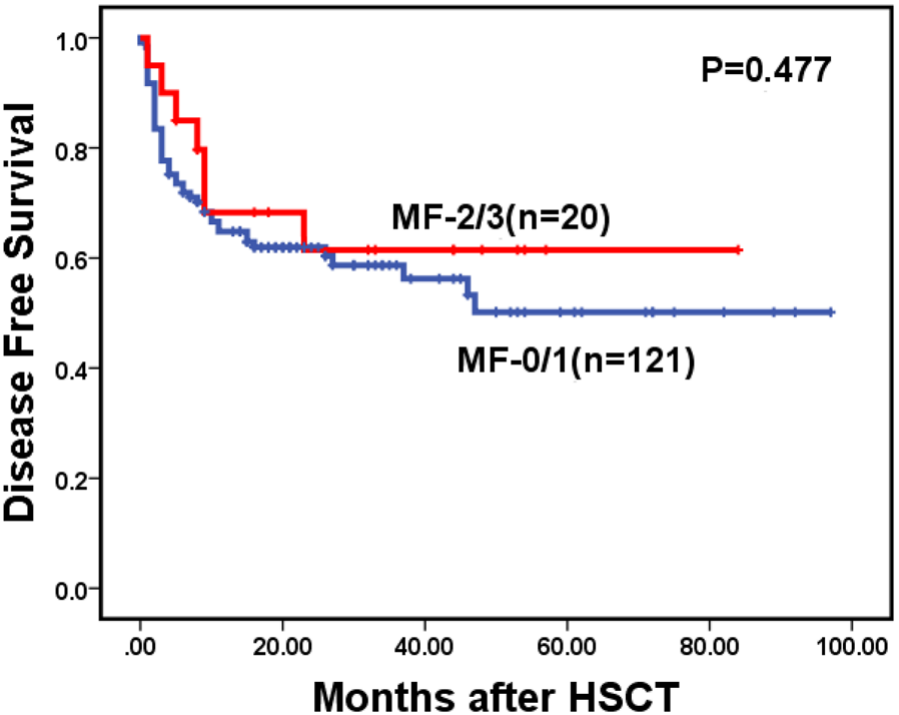
Disease-free survival of MDS patients with allo-HSCT classified by MF (MF-0/1 VS. MF-2/3)

**Fig. 9:**
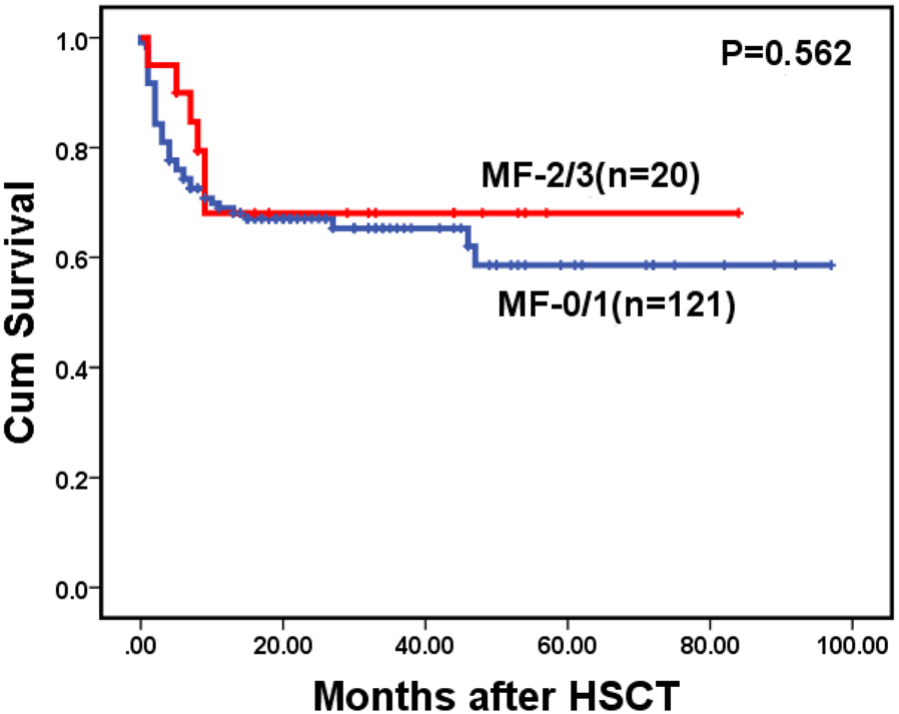
Overal survival after allo-HSCT of MDS patients classified by MF (MF-0/1 VS. MF-2/3)

Of 141 patients who underwent transplantation, 19 patients experienced relapse at a median time of 26 months (range: 1–97) post-transplantation, including 16 cases in the MF-0/1 and 3 in the MF-2/3 group. The cumulative incidence of relapse at 2 years post-transplantation was 12.9% (95% CI: 7.8–19.4%), along with 12.0% (95% CI: 6.9–18.7%) in the MF-0/1 and 16.8% (95% CI: 3.7–38.1%) in the MF-2/3 group (*P* = 0.910) (Fig. 6). By the time of follow-up, 48 patients died after transplantation, including 10 patients died of relapse and 38 patients died without relapse. The cumulative incidence of non-relapse mortality (NRM) at 2 years post-transplantation was 25.3% (95% CI: 18.4–32.9%), along with 26.0% (95% CI: 18.5–34.2%) in the MF-0/1 and 21.7% (95% CI: 6.4–42.8%) in the MF-2/3 group (*P* = 0.441) (Fig. 7). The 2-year DFS post-transplantation was 61.8% (95% CI: 53.6–70.0%), along with 62.0% (95% CI: 53.2–70.8%) in the MF-0/1 and 61.5% (95% CI: 40.3–82.7%) in the MF-2/3 group (*P* = 0.477) (Fig. 8). The 2-year OS post-transplantation was 65.8% (95% CI: 61.6–74.0%), along with 65.3% (95% CI: 56.3–74.3%) in the MF-0/1 and 68.1% (95% CI: 46.9–89.3%) in the MF-2/3 group (*P* = 0.562) (Fig. 9).

In univariate and multivariate analysis, complex karyotype was an unfavorable factor for relapse (HR 3.51, *P* = 0.012 and HR 4.16, *P* = 0.006, respectively), DFS (HR 2.24, *P* = 0.012 and HR 2.16, *P* = 0.020, respectively), and OS (HR 2.28, *P* = 0.014 and HR 2.47, *P* = 0.009, respectively) post-transplantation (Table 7).

**Table 7:** Risk features of myelofibrosis in MDS patients underwent HSCT by Cox regression analysis (n = 141)

Variable	Relapse		NRM		DFS		OS	
	Univariate	Multivariate	Univariate	Multivariate	Univariate	Multivariate	Univariate	Multivariate
		*P*		*P*		*P*		*P*
		HR (95% CI)		HR (95% CI)		HR (95% CI)		HR (95% CI)
Age	0.988	–	0.169	–	0.21	–	0.21	–
	1.00 (0.96–1.04)		1.02 (0.99–1.05)		1.02 (0.99–1.04)		1.02 (0.99–1.04)	
Sex		–		–		–		–
Male	Ref.		Ref.		Ref.		Ref.	
Female	0.566		0.304		0.245		0.319	
	0.76 (0.30–1.93)		0.70 (0.36–1.38)		0.72 (0.42–1.25)		0.74 (0.41–1.34)	
Cytogenetics								
Non–complex karyotypes	Ref.	Ref.	Ref.	Ref.	Ref.	Ref.	Ref.	Ref.
Complex karyotypes	0.012	0.006	0.328	0.347	0.012	0.020	0.014	0.009
	3.51 (1.32–9.34)	4.16 (1.51–11.45)	1.51 (0.66–3.46)	1.51 (0.64–3.55)	2.24 (1.19–4.20)	2.16 (1.13–4.12)	2.28 (1.18–4.42)	2.47 (1.25–4.90)
BM blasts at HSCT								
≤ 5%	Ref.	Ref.	Ref.	Ref.	Ref.	Ref.	Ref.	Ref.
> 5%	0.349	0.253	0.343	0.245	0.782	0.773	0.763	0.73
	1.80 (0.53–6.20)	2.08 (0.59–7.31)	0.72 (0.36–1.43)	0.66 (0.33–1.33)	0.92 (0.51–1.66)	0.92 (0.51–1.66)	0.91 (0.48–1.72)	0.89 (0.47–1.69)
Myelofibrosis								
MF = 0/1	Ref.	Ref.	Ref.	Ref.	Ref.	Ref.	Ref.	Ref.
MF = 2/3	0.964	0.914	0.986	0.286	0.459	0.396	0.569	0.444
	0.97 (0.28–3.34)	1.07 (0.31–3.76)	1.02 (0.13–8.15)	0.56 (0.20–1.62)	0.74 (0.34–1.64)	0.71 (0.32–1.58)	0.78 (0.33–1.84)	0.71 (0.30–1.70)
Time to HSCT from diagnosis								
≤ 6 months	Ref.	Ref.	Ref.	Ref.	Ref.	Ref.	Ref.	Ref.
> 6 months	0.77	0.888	0.56	0.709	0.952	0.678	0.741	0.526
	0.86 (0.31–2.39)	1.08 (0.38–3.08)	0.63 (0.13–3.02)	1.15 (0.56–2.34)	1.02 (0.58–1.80)	1.13 (0.63–2.04)	1.11 (0.60–2.04)	1.23 (0.65–2.31)
Previous therapy for MDS								
No cytoreductive treatments	Ref.	Ref.	Ref.	Ref.	Ref.	Ref.	Ref.	Ref.
Cytoreductive treatments	0.468	0.327	0.337	0.642	0.902	0.856	0.794	0.627
	1.41 (0.56–3.60)	1.61 (0.62–4.19)	1.97 (0.49–7.89)	0.86 (0.45–1.64)	0.97 (0.57–1.63)	1.05 (0.62–1.79)	1.08 (0.61–1.91)	1.16 (0.64–2.07)
Donor source								
HID	Ref	–	Ref	–	Ref	–	Ref	–
MUD	0.806		0.277		0.709		0.903	
	0.77 (0.09–6.50)		2.49 (0.48–12.81)		0.82 (0.28–2.37)		1.07 (0.37–3.13)	
MSD	0.393		0.374		0.646		0.717	
	1.54 (0.57–4.11)		0.48 (0.09–2.45)		0.88 (0.51–1.51)		0.90 (0.50–1.62)	
Stem cell source								
PB	Ref	–	Ref	–	Ref	–	Ref	–
PB + BM	0.325		0.628		0.899		0.666	
	1.60 (0.63–4.07)		0.85 (0.45–1.62)		1.03 (0.61–1.74)		1.13 (0.64–2.00)	

*NRM* non-replase mortality, *RI* relapse incidence, *DFS* disease-free survival, *OS* overall survival (after allo-HSCT), *MF* myelofibrosis

## Discussion

In this retrospective study, we analyzed the biological characteristics of MDS patients with different degrees of MF and assessed the impact of MF on the prognosis of these patients. The results showed that patients with MF-2/3 have more complex karyotypes and lower ORR of cytoreduction. Among patients without allo-HSCT, patients with MF-2/3 have shorter survival than those with MF-0/1. Fortunately, allo-HSCT might overcome the adverse factor of MF.

MF is considered a poor prognostic factor in patients with MDS. Our results indicated that the 2-year OS was only 19.2% in the MF-2/3 group if allo-HSCT was not performed. Why MF affects the survival of MDS patients remains unclear. Buesche G [6] considered some correlation between MF and multilineage dysplasia, excess of blasts, and increased risk of early bone marrow failure. Melody M [20] reported that more patients with MF-3 were classified as MDS with excess of blasts and with higher IPSS-R risk categories than those with MF-0/1/2. Our study found complex karyotypes were more common in the MF-2/3 than in the MF-0/1 group, which was consistent with the previous study [20]. Moreover, some studies suggested that poor-risk cytogenetics is often accompanied by worse survivals in MDS [2, 31–33], which might partially explain the shorter OS observed in the MF-2/3 group. In line with the previous studies [3, 34], our data showed that MF did not add additional risk to leukemia transformation. However, we found the median transformation time was shorter in the MF-2/3 than in the MF-0/1 group. Besides, patients who failed to respond to HMA treatment often display significantly worse OS [35, 36], and a lower ORR of cytoreduction was observed in the MF-2/3 compared with the MF-0/1 group in our study. These factors all might be the causes of the poor prognosis in the MF-2/3 group.

It remains controversial whether allo-HSCT can overcome the adverse factor of MF. Some studies suggested that MF did not affect the outcomes of allo-HSCT in the patients with MDS [19, 37], while others indicated that MF might be associated with lower engraftment rate, higher relapse, and NRM [5, 38, 39]. In this study, we observed a delay in neutrophil and platelet reconstruction in the MF-2/3 group. Still, there was no difference in the cumulative incidence of neutrophil and platelet engraftment achieved at day + 30 between the two groups. This observation might be related to the MAC regimens and ATG because some studies reported that MAC regimens or receiving ATG along with a conditioning regimen were protective against graft failure [40, 41]. Moreover, the addition of bone marrow stem cells and the relatively large number of transfused cells might also partially explain the high engraftment rate in this study.

Regarding the relapse incidence, Kröger N, et al. reported a significantly higher risk of relapse in the group with severe fibrosis, but only in univariate analysis [5]. Our study found complex karyotype, not MF, that affected the relapse post-transplantation. Discrepant results had been reported regarding the impact of MF on NRM, with some studies suggesting no influences [19], while other studies reported that higher degrees of fibrosis in the marrow were paralleled by a greater likelihood of fibrosis in other organs (e.g., liver and lungs), making these organs more susceptible to regimen-related toxicity after HSCT and thus increasing NRM [39]. In our study, we did not observe a higher NRM in the MF-2/3 group. Additionally, our results also demonstrated that MF did not influence the incidence of acute or chronic GVHD. Considering the above factors, it is not surprising that patients with MF-2/3 can achieve the comparable DFS and OS of patients with MF-0/1.

In summary, patients with MF-2/3 have more complex karyotypes and lower ORR of cytoreduction in MDS. Among patients without allo-HSCT, patients with MF-2/3 have a worse prognosis than those with MF-0/1. However, the adverse impact of MF on prognosis may be overcome by allo-HSCT.

## Limitations

Inevitably, there were limitations to our study. First, the sample size of patients with MF-2/3 who received HSCT was relatively small. Second, we lacked the data of MF in patients after HSCT, so we could not analyze the impact of HSCT on MF in BM. Finally, because retrospective nature of analyses and subjects enrolled in this study were only dependent on hospitalized patients, there was an inevitable selection bias in our study.

## Data Availability

All data generated or analyzed during this study are included in this manuscript.
